# Leveraging systems biology for predicting modulators of inflammation in patients with COVID-19

**DOI:** 10.1126/sciadv.abe5735

**Published:** 2021-02-03

**Authors:** Sascha Jung, Ilya Potapov, Samyukta Chillara, Antonio del Sol

**Affiliations:** 1 Computational Biology Group, CIC bioGUNE-BRTA (Basque Research and Technology Alliance), Bizkaia Technology Park, Derio 48160, Spain.; 2 Computational Biology Group, Luxembourg Centre for Systems Biomedicine (LCSB), University of Luxembourg, L-4362 Esch-sur-Alzette, Luxembourg.; 3 IKERBASQUE, Basque Foundation for Science, Bilbao 48013, Spain.

## Abstract

Dysregulations in the inflammatory response of the body to pathogens could progress toward a hyperinflammatory condition amplified by positive feedback loops and associated with increased severity and mortality. Hence, there is a need for identifying therapeutic targets to modulate this pathological immune response. Here, we propose a single cell–based computational methodology for predicting proteins to modulate the dysregulated inflammatory response based on the reconstruction and analysis of functional cell-cell communication networks of physiological and pathological conditions. We validated the proposed method in 12 human disease datasets and performed an in-depth study of patients with mild and severe symptomatology of the coronavirus disease 2019 for predicting novel therapeutic targets. As a result, we identified the extracellular matrix protein versican and Toll-like receptor 2 as potential targets for modulating the inflammatory response. In summary, the proposed method can be of great utility in systematically identifying therapeutic targets for modulating pathological immune responses.

## Teaser

A systems biology approach predicts immunomodulators in diseases characterized by pathological inflammation, such as COVID-19.

## INTRODUCTION

Inflammation is a key defense mechanism to pathogenic factors, such as infection, chemical substances, or tissue injury, which is mediated by tissue resident and circulating cells recruited from the blood through the establishment of chemokine gradients. To modulate the level of inflammation, these cells release cytokines to communicate with each other and activate cell type–specific functions necessary to clear the pathogenic factor. For instance, phagocytic activity removes cellular debris and pathogens, thereby suppressing inflammation through the release of anti-inflammatory cytokines, such as interleukin-10 (IL-10) and transforming growth factor–β (*1*). Typically, the immune response is tightly controlled to minimize tissue injury and restore homeostasis. However, in case the pathogenic factor cannot be cleared, an acute inflammatory response progresses toward a hyperinflammatory condition, commonly referred to as “cytokine storm,” due to an excessive release of cytokines and an accumulation of immune cells in the tissue.

Recently, a novel, highly contagious coronavirus [severe acute respiratory syndrome coronavirus 2 (SARS-CoV-2)] has been identified as the causative pathogen of an ongoing outbreak of viral pneumonia [coronavirus disease 2019 (COVID-19)]. Accumulating evidence suggests that patients with severe symptomatology of COVID-19 develop such a hyperinflammatory immune response, similar to other respiratory infectious diseases such as influenza infection (*2*). Although initial studies have begun to arise in which the immune status of patients is assessed (*3*, *4*), the molecular mechanisms underlying the hyperinflammatory response of patients with more severe symptoms are not readily known. Hence, the present challenge is how to mitigate this cytokine storm while not impairing viral clearance.

Cytokine storms are most often characterized by high levels of tumor necrosis factor (TNF), interferons, and IL-6, as well as the accumulation of immune cells in the tissue (*5*–*7*). However, the composition of proinflammatory ligands involved in cytokine storms depends on the viral pathogen. For instance, in patients with SARS, hyperinflammation is characterized by high levels of interferon-γ (IFNG), IL-18, and lymphotoxin-α (LT-α), whereas patients with severe influenza infections present high levels of TNF, IL-6, and IL-1 (*6*, *7*). Moreover, cytokine storms are mediated by interactions between immune and nonimmune cells, which form positive feedback loops responsible for amplifying and maintaining the immune response. These loops are composed of cell-cell interactions for which incoming signals induce outgoing signals in each participating cell population. For example, in case of influenza infection, a positive feedback loop involves the production of IL-1 and TNF by macrophages in response to granulocyte-macrophage colony-stimulating factor released by nonhematopoietic cells (*8*). In the case of Epstein-Barr virus infection, the hyperinflammatory response is amplified by multiple, interconnected positive feedback loops between dendritic cells, CD8^+^ T cells, natural killer (NK) cells, and macrophages that involve several proinflammatory cytokines, including IL-1, IL-6 and IL-18, IFNG, and TNF (*9*). However, in general, positive feedback loops responsible for the amplification of proinflammatory signals are largely unknown. Furthermore, they differ in the participating cell populations, as well as in the involved inflammatory molecules depending on the tissue type and the viral infection. Consequently, it is critical to characterize the positive feedback loops amplifying and maintaining the hyperinflammatory immune response to develop therapeutic strategies for selectively disrupting cell-cell interactions underlying these conditions.

The development of strategies for modulating the immune response typically relies on the identification of biomarkers as therapeutic targets or the large-scale in vitro screening of compounds. While these approaches have led to the identification of immunomodulatory compounds for a diverse array of underlying diseases (*10*), they are laborious and resource intensive or, as in the case of biomarkers, mostly not efficacious, which impedes the design of novel therapeutic strategies for modulating inflammation. The upsurge of single-cell sequencing technologies has enabled the analysis of multiple cell populations in a tissue at an unprecedented resolution and permits the development of computational methods that could identify immunomodulatory therapeutic targets and compounds inhibiting them. To date, great efforts have been devoted to the discovery of biomarkers and drug-target interactions (*11*–*13*). However, to our knowledge, no computational method for predicting immunomodulatory target proteins that could alleviate pathologic dysregulations of the physiological immune response exists.

To address this issue, we present a single cell–based computational method for the systematic prediction of protein targets to modulate the inflammatory response. In particular, given two tissue-specific single-cell profiles of a dysregulated immune response and a physiological control, our method infers the functional cell-cell communication networks of both conditions by integrating a collection of 1756 extracellular receptor-ligand interactions from more than 6000 protein-protein interactions (PPIs) with intracellular signaling and gene regulatory networks. Once the functional cell-cell communication network has been inferred, to construct positive feedback loops, the method searches for those interactions causing the expression of ligands secreted by this cell population. As a plausible therapeutic strategy to modulate the pathological immune response, we propose to target these positive feedback loops. In this regard, our method simulates the effect of perturbing receptor-ligand interactions, prioritizes genes specifically disrupting pathological while preserving physiological feedback loops, and links them to compounds targeting them. To validate our method for predicting immunomodulatory target proteins and to demonstrate its general applicability, we applied it to 12 disease pathologies characterized by hyperinflammatory or chronic inflammation and were able to validate 90% of the predicted target proteins. Similar to a hyperinflammatory immune response, the released cytokines under chronic inflammatory conditions are highly promiscuous, depend on the causative pathogenic factor and the affected tissue, and form positive feedback loops to amplify and maintain an elevated immune response (*5*–*7*, *14*).

Last, we applied our method to a recently published dataset of bronchoalveolar lavage fluid from patients with mild and severe symptomatology of COVID-19 (*15*). Our method revealed that the hyperinflammatory response in patients with severe symptomatology is maintained by interconnected feedback loops involving multiple proinflammatory cytokines and extracellular matrix proteins as well as the reprogramming of the anti-inflammatory immune response by IL-10 into a proinflammatory phenotype. In addition, we demonstrate that T cell recruitment is impaired because of the disruption of a feedback mechanism between T cells, secretory epithelial cells, and macrophages, which explains the defective viral clearance in patients with severe symptomatology. Last, computational perturbation of genes involved in causal feedback loops identified versican (VCAN), an extracellular matrix glycoprotein, and Toll-like receptor 2 (TLR2) as novel target proteins for alleviating the hyperinflammatory response in patients with COVID-19 with severe symptomatology. Thus, in summary, we believe that this method can be of great utility in the systematic identification of therapeutic targets for modulating pathological immune responses.

## RESULTS

### Elucidating positive feedback loops in cell-cell communication networks for predicting immunomodulatory target proteins and compounds in diverse disease pathologies

**Fig. 1:**
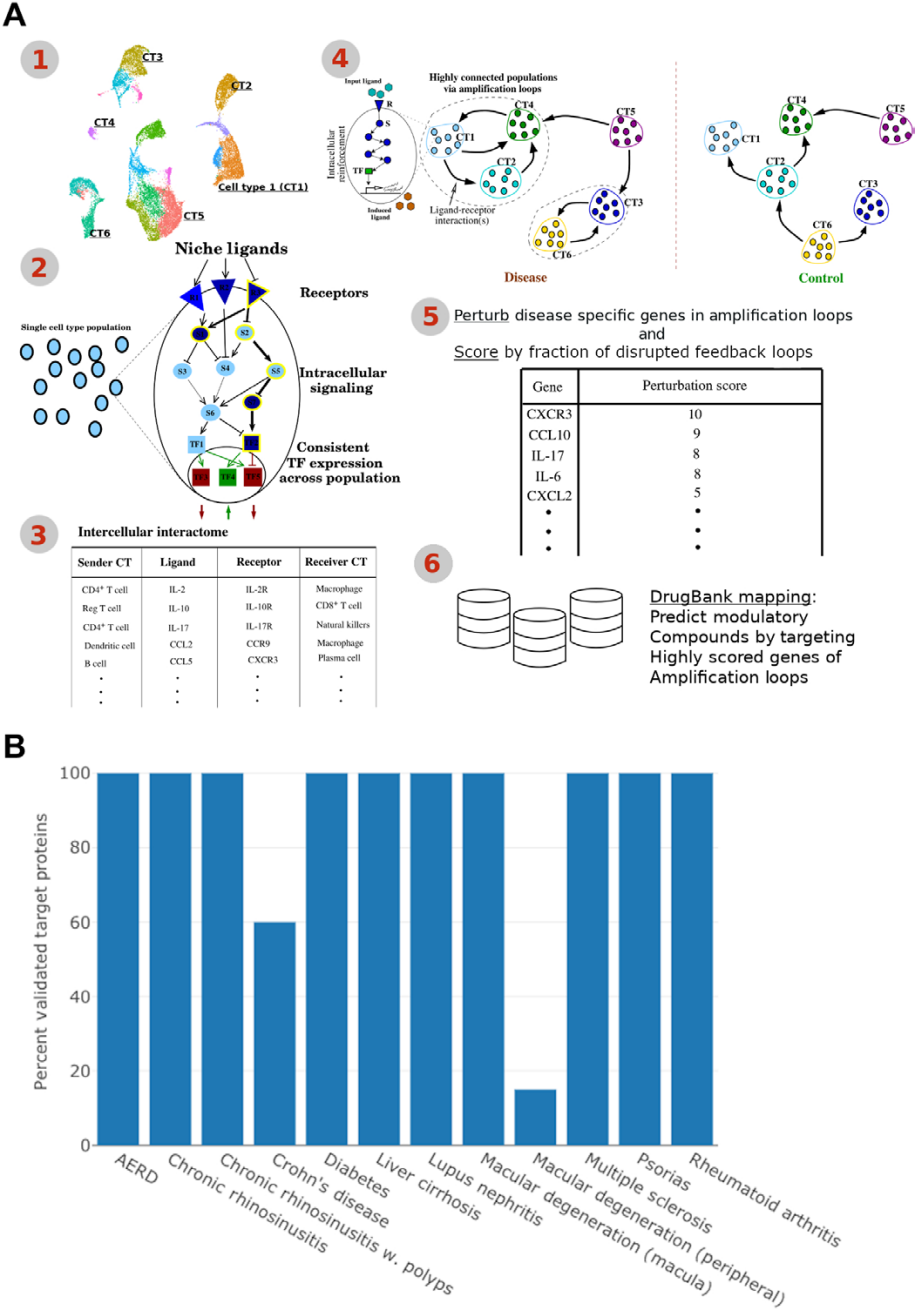
Method overview and validation in 11 disease pathologies. (**A**) The method workflow consists of the following steps: (1) single-cell RNA sequencing (RNA-seq) data with cell type (CT) annotations as an input; (2) each population is examined for the persistent signaling flow from receptors via signaling molecules (pathways) to the phenotype-determining TFs; (3) the phenotype-maintaining receptors form the final interactome along with their cognate ligands from other populations; (4) the interactome is further examined for presence of the positive feedback loops, extracellular (from ligands to receptors) and intracellular (from receptors via signaling pathways to ligands) paths that maintain each other by forming closed loops; (5) disease-specific ligands and receptors that are responsible for the formation of the feedback loops and are ranked on the basis of their ability to disrupt the positive feedback loops unique to pathological immune responses; (6) high-scoring genes are mapped to DrugBank for identifying inhibitory compounds. (**B**) Percentage of predicted target proteins with literature evidence per example. Except in peripheral age-related macular degeneration (15%) and Crohn’s disease (60%), all predicted target genes are validated. AERD, aspirin-exacerbated respiratory disease.

Because of the well-established effect of cellular feedback in the control of the inflammation, we hypothesized that the positive feedback loops established between immune and nonimmune cells are responsible for amplifying and maintaining an elevated immune response. To detect these feedback loops, we first set out to reconstruct the ligand-receptor–mediated cell-cell communication network within a tissue (Fig. 1A). In this regard, we manually curated more than 6000 PPIs between receptors and ligands and identified 1756 experimentally validated, extracellular interactions (table S1). Next, we integrated this set of high-confidence interactions with intracellular signaling and gene regulatory networks to infer the cell-cell communication network and positive feedback loops (Fig. 1A). In particular, we first selected transcription factors (TFs) whose expression is preserved across cells in each population and, second, identified the active receptors regulating them using a Markov chain model that assess signal transduction probabilities. Last, cognate ligands were identified for each active receptor, which are expressed in more than 5% of the secreting cell population. Only statistically significant ligand-receptor interactions remained in the final cell-cell communication network of each condition, assessed by the strength of each interaction compared to all potential interactions. Once the functional cell-cell communication network has been inferred, the method searches for those interactions causing the expression of ligands secreted by the receiving population by warranting the existence of a sustained regulatory path from the receptor to the ligand (see Materials and Methods for details). Positive feedback loops are lastly established by combining extracellular and intracellular ligand-receptor interactions. A detailed description of the methodology can be found in Materials and Methods.

To predict immunomodulatory target proteins, we propose to score genes by their ability to disrupt positive feedback loops when perturbed. Namely, given the positive feedback loops underlying pathological and physiological immune responses, each gene is synthetically removed from the feedback loops unique to the pathological condition. The fraction of ligands and receptors that are removed by this synthetic perturbation serves as a score for the gene. We score only those feedback loops and genes that differ between the pathological and corresponding physiological conditions. Last, the method extracts information from DrugBank (*16*) to select drug candidates targeting the highly scored genes (see Materials and Methods for details).

We set out to validate our approach for identifying immunomodulatory proteins and to demonstrate its general applicability by applying it to a vast array of diseases that are characterized by a pathological immune response. In particular, the considered pathologies include autoimmune diseases, such as lupus nephritis, chronic diseases, such as Crohn’s disease and type II diabetes, as well as allergic conditions, such as aspirin-exacerbated respiratory disease (table S2). As a result, we identified a median of two top-ranking target proteins having the highest scores, with two notable exceptions. In particular, in case of liver cirrhosis and age-related macular degeneration samples from the peripheral eye region, our method identified 15 and 20 target proteins, respectively. Validation with previous literature revealed evidence for, on average, 90% of the predicted immunomodulatory proteins (Fig. 1B and table S3). Nevertheless, only 60 and 15% of proteins predicted for Crohn’s disease and peripheral age-related macular degeneration samples could be validated, respectively.

### Mild and severe COVID-19 manifestations have distinct transcriptional profiles

**Fig. 2:**
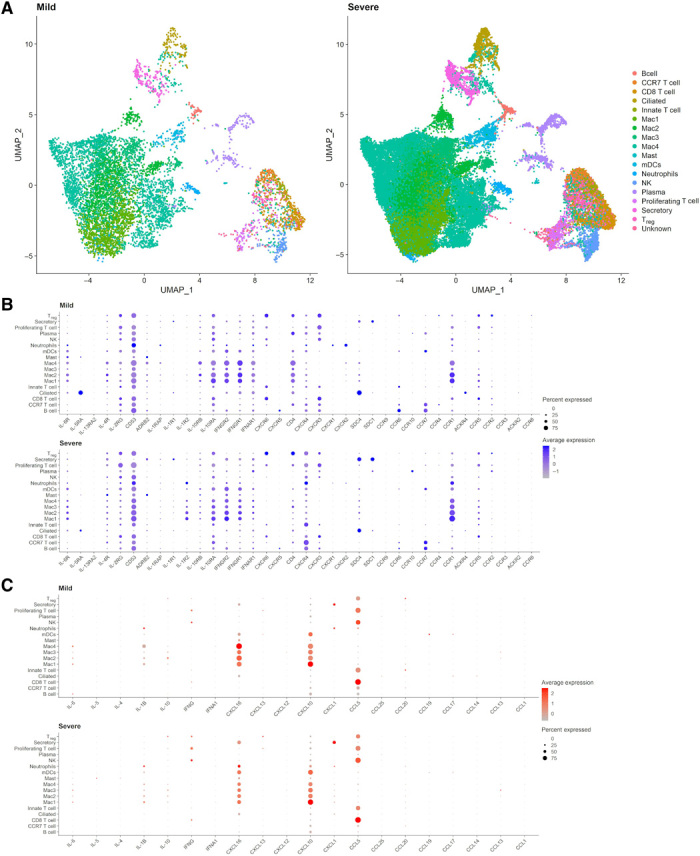
Transcriptional characterization of bronchoalveolar lavage fluid samples of patients with COVID-19. (**A**) UMAP (uniform manifold approximation and projection) representation of integrated samples from patients with mild (left) and severe (right) symptoms. T_reg_, regulatory T cells. (**B**) Expression of selected pro- and anti-inflammatory cytokine and chemokine receptors per identified cell type in patients with mild (top) and severe (bottom) symptoms. (**C**) Expression of selected pro- and anti-inflammatory cytokines and chemokines per identified cell type in patients with mild (top) and severe (bottom) symptoms.

We sought to perform an in-depth case study and analyzed recently published single-cell RNA sequencing (RNA-seq) data of nine Chinese patients with COVID-19 with mild and severe symptomatology and three healthy individuals (*15*). For each group, we aggregated data of different patients presenting with mild and severe symptoms and healthy individuals into a single representative sample of each condition and clustered cells to identify cell types using known sets of markers (*15*). We identified 15 common cell types for both groups: B cells, CCR7^+^ T cells, CD8^+^ T cells, proliferating T cells, innate T cells, regulatory T cells, four subpopulations of macrophages (Mac1 to Mac4), ciliated cells, secretory cells, plasma cells, myeloid dendritic cells (mDCs), and NK cells. In addition, neutrophils and mast cells were uniquely identified in the severe cases. (Fig. 2A and fig. S1A).

Before reconstructing the cell-cell communication networks underlying both conditions, we performed differential expression analysis of each cell type under both conditions. As a result, we observed that the mild and severe groups significantly differ in the levels of expression of inflammatory molecules (Fig. 2, B and C). For instance, chemokines, including CCL2 (CC chemokine ligand 2), CCL3, CCL4, and CCL8, are significantly up-regulated in nearly all cell types of patients with severe symptoms. In contrast, chemokines, such as CXCL2 (C-X-C motif chemokine ligand 2), CXCL3, and CXCL9, together with their receptor CXCR4 (C-X-C motif chemokine receptor 4), are up-regulated in specific cell types such as in macrophages. In addition, certain pro- and anti-inflammatory cytokines and their receptors have elevated levels under the severe condition, such as IL-6 and its receptor in macrophages, IL-18 in ciliated cells, IL-4R (IL-4 receptor) and IL-7R. On the contrary, among the up-regulated cytokines under the mild condition are IL-18 in macrophages, CCL20 in innate and regulatory T cells, CXCR6 in NK cells, and VASP (vasodilator-stimulated phosphoprotein) protein in certain macrophage populations and T cells. Known anti-inflammatory cytokines do not show consistent differential expression. Thus, for example, the expression of IL-10 is marginally elevated in Mac3 and regulatory T cell populations of the severe cases, whereas the expression level of IL-1R2 (IL-1 decoy receptor) and IL-1 receptor antagonist (IL-1RN) is elevated in all macrophage populations and mDCs. In contrast to patients with COVID-19, samples obtained from healthy individuals do largely not display expression of cytokines and chemokines (fig. S1, B and C). Notably, CXCL16, the cognate ligand of CXCR6, is expressed in most cells and cell types, which suggests that with increasing severity, CXCL16 expression is substantially decreased (fig. S1D).

### Disease severity is characterized by specific cell-cell communication networks

**Fig. 3:**
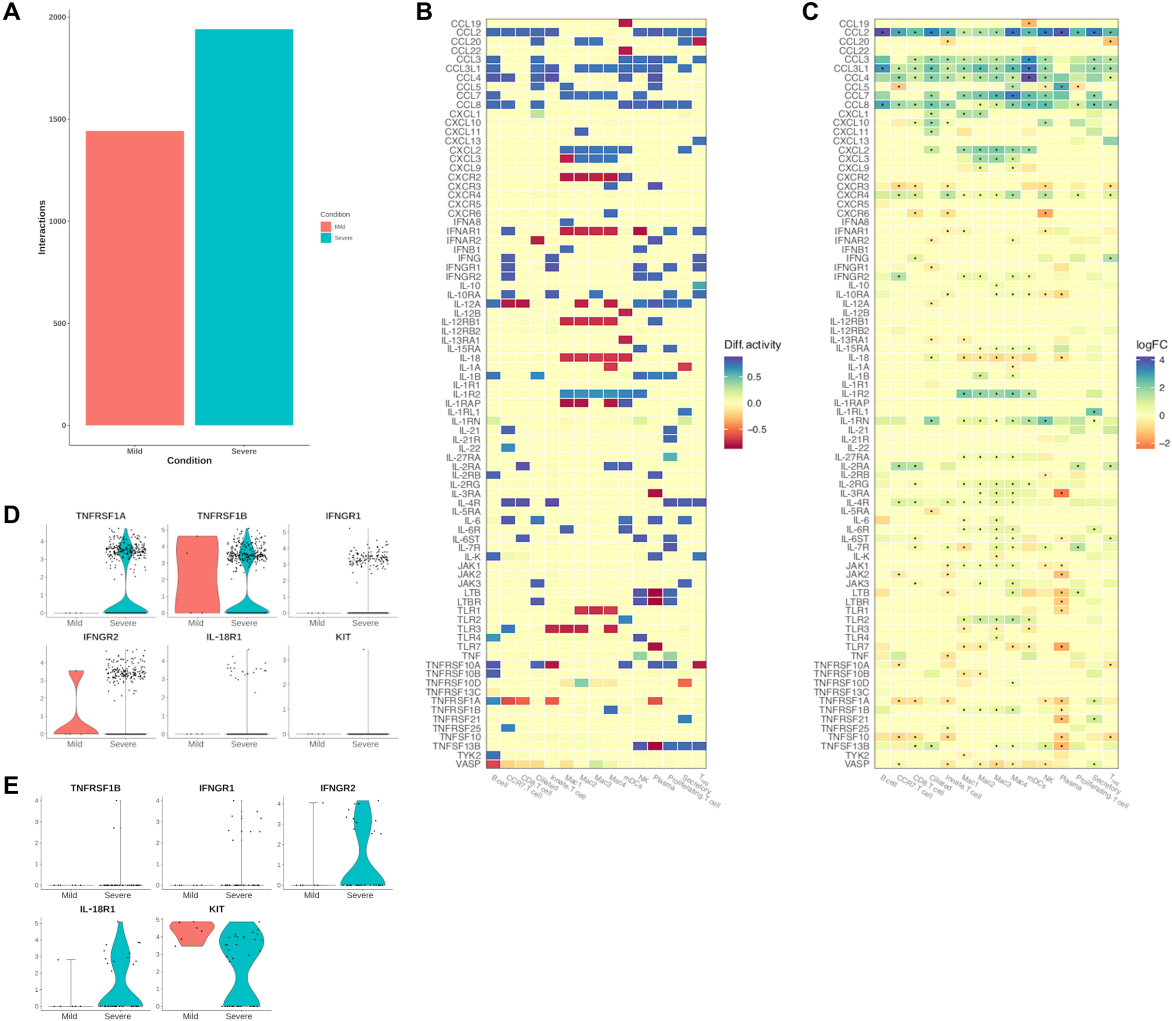
Cell-cell communication network analysis of patients with COVID-19. (**A**) Number of interactions detected in each cell-cell communication network. (**B**) Differential signaling activity of ligands and receptors involved in immune response based on SigHotSpotter. (**C**) Differential expression of ligands and receptors involved in immune response. Dots represent significant differences [adjusted *P* < 0.05, obtained by MAST analysis, Seurat implementation (*34*, *35*)]. (**D** and **E**) Expression of receptors characteristic for neutrophils (D) and mast cells (E) under both conditions. JAK1, Janus kinase 1; TYK2, tyrosine kinase 2.

To elucidate the intercellular interactions maintaining the differences in the expression of ligands and receptors, we use our method to reconstruct the cell-cell communication networks underlying mild and severe disease courses and observed vast differences, which is signified by a 33% increase in interactions in severe cases (Fig. 3A). Despite the observed increase in cell-cell interactions, the number of unique molecules per condition that are involved in extracellular signaling remains low. The severe condition is characterized by interactions involving the proinflammatory cytokines IL-1B, IL-1A, TNF superfamily member 4 (TNFSF4), and thymic stromal lymphopoietin. In contrast, the mild condition is distinguished by ligands promoting lymphocyte migration, such as CXCL13, CCL24, CCL7 and CCL20 as well as cell-adhesion mediators including tenascin C (TNC). The unique interactions involving these ligands cannot be fully explained by differential expression analysis. For instance, IL-21 nor its receptor IL-21R is differentially expressed in any cell type. However, a significant difference in the signal transduction probabilities of IL-21R can be observed in proliferating T cells, which underscores the advantages of our approach (Fig. 3, B and C).

Next, we investigated whether unique interactions can be explained by differences in the composition of cell populations between the two conditions. While the relative proportion of most cell populations in the cell-cell communication networks differs by at most 1%, neutrophils and mast cell interactions are specific to severe disease courses. Consistent with their role in the induction of inflammation (*17*, *18*), these cells release proinflammatory cytokines, such as TNF, IL-1B, IL-18, and oncostatin M, responsible for the positive regulation of nuclear factor κB of activated B cells. Further, neutrophils and mast cells express receptors of proinflammatory cytokines including TNF receptor 1, interferon-γ receptor 2, IL-18R, and the mast cell growth factor receptor KIT, which suggest their involvement in proinflammatory feedback mechanisms (Fig. 3, D and E).

### Distinct cellular feedback mechanisms control the inflammatory response in patients with mild and severe COVID-19

Although a plethora of clinical trials has been performed or is currently ongoing that probed the efficacy of drugs in the treatment of patients presenting with a hyperinflammatory immune response, no effective treatment has been found. Therefore, we sought to apply our methodology to identify proteins that could be targeted for modulating the immune response in patients with severe symptomatology of the COVID-19. In this regard, we first identified the positive feedback loops underlying mild and severe cases, respectively, and detected three groups (fig. S2). The first group signifies the immune response common to patients with severe and mild symptomatology and is characterized by feedback between pro- and anti-inflammatory cytokines predominantly released by macrophages and dendritic cells (table S4). In particular, proinflammatory cytokine signaling by TNF and IFNG is fine-tuned by anti-inflammatory ligands, such as IL-10 and the TNF receptor antagonist progranulin (GRN). In contrast, the second and third groups contain interactions unique to patients with mild and severe pathologies, respectively. In contrast to patients with COVID-19, healthy individuals display only a single positive feedback loop between fibronectin (FN1) and plasminogen activator, urokinase receptor (PLAUR) not involving any cytokines or chemokines, as expected from the transcriptional analysis. To gain insights into the individual stabilizing mechanisms of the immune response in patients with mild and severe symptomatology, we set out to characterize the feedback mechanisms underlying both groups.

### Promotion of lymphocyte survival and recruitment in patients with mild symptomatology

Analysis of the cell-cell communication landscape of mild cases reveals two distinct clusters of positive feedback loops unique to this condition. The first cluster consists of B cells secreting macrophage inhibitory factor (MIF), which is sensed in an autocrine and paracrine manner by human leukocyte antigen class II histocompatibility antigen gamma chain (CD74). This interaction induces B cell survival and proliferation through activation of the PI 3-kinase/Act pathway, thus, rescuing them from apoptosis (*19*). The absence of autocrine and paracrine MIF stimulation of B cells in patients with severe symptomatology suggests an impaired B cell response to SARS-CoV-2 due to increased apoptosis.

**Fig. 4:**
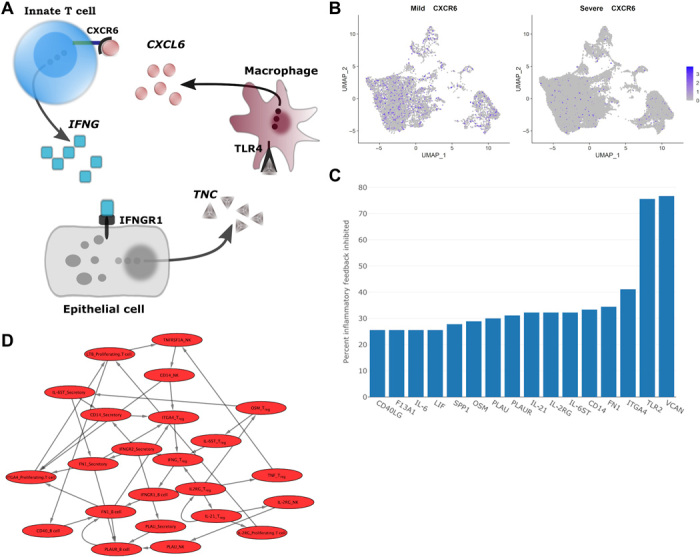
Prediction of immunomodulatory proteins in patients with severe COVID-19. (**A**) A feedback loop between T cells, secretory epithelial cells, and macrophages maintain T cell recruitment in patients with mild symptoms. (**B**) Expression of CXCR6 across cell populations in mild and severe cases. (**C**) Predicted scores for each potential target protein expressed as the percentage of inflammatory feedback loops inhibited after computational perturbation. (**D**) Uninterrupted feedback loops after computational inhibition of VCAN in patients with severe symptoms.

In contrast to the first cluster, the second group of interconnected feedback loops involves a chemotactic interaction between CXCL16 and CXCR6. In particular, CXCL16 and CXCR6 belong to a causal feedback loop between T cells, secretory epithelial cells, and macrophages (Fig. 4A). According to our model, CXCR6 activates IFNG release in innate T cells, which is received by IFNGR1 (IFNG receptor 1) in secretory cells. In turn, IFNG induces the expression of TNC, an extracellular glycoprotein up-regulated in infected tissues, which binds to TLR4 and results in secretion of CXCL16. Mechanistically, the interaction between CXCL16 and CXCR6 is necessary for the recruitment of T lymphocytes to infected tissues. The absence of this interaction together with a 56% decline in innate T cells expressing CXCR6 demonstrates the impaired innate T cell recruitment in patients with severe symptomatology (Fig. 4B). In summary, the feedback loops unique to mild disease courses contribute to survival and recruitment of B and T lymphocytes required for viral clearance.

#### Hyperinflammation is maintained in patients with severe symptomatology

In addition to mild disease cases, we analyzed the causal feedback loops identified in patients with severe symptomatology to characterize distinct patterns in the immune response. Severe COVID-19 cases are known to develop a hyperinflammatory response to SARS-CoV-2, which is characterized by an excess of proinflammatory cytokine secretion (*20*). To elucidate the mechanism that maintains the excessive release of cytokines in severe cases, we first performed a topological analysis of the interconnected feedback loops and assessed differences in network stability between mild and severe cases. In contrast to mild cases, we observed a threefold increase in the interconnectivity of the feedback loops (density, 0.219 and 0.072), which can be attributed to the establishment of redundancy in receptor activation. While, in mild cases, each receptor is activated by a median of one ligand, a median of three ligands activates each receptor in severe cases according to our model (one-sided Wilcoxon rank sum test, *P* = 0.009). In addition, the number of induced ligands per receptor significantly decreases (one-sided Wilcoxon rank sum test, *P* = 0.002), which implies that more cellular populations participate in the formation of causal feedback loops in severe cases compared to mild disease courses. In summary, patients with severe symptomatology show a significant redundancy in the activation of receptors and intracellular signaling cascades. Because of the involvement of an increased number of cellular populations in the activation of these receptors, a stable feedback regulation is established that is robust to fluctuations in ligand secretion.

Next, we sought to determine whether this stable feedback regulation maintains the hyperinflammatory immune response by secretion of proinflammatory cytokines. In contrast to patients with mild symptoms, we observed causal feedback loops involving an array of proinflammatory cytokines, namely, TNF, IFNG, LT-β, IL-27, IL-6, IL-15, and IL-18. Moreover, these loops contain the extracellular matrix proteins FN1, VCAN, and PLAU, which suggests the induction of proinflammatory ligand secretion by cell adhesion. In agreement with previous reports, our model predicts that these genes induce the expression of the proinflammatory cytokines IL-15 and IFNG (*21*, *22*). Therefore, the hyperinflammatory condition in patients with severe COVID-19 symptomatology is maintained by the redundant secretion of proinflammatory cytokines and signaling through extracellular matrix proteins that reinforces their induction.

#### IL-10 induces the expression of proinflammatory cytokines in macrophages

In addition to the maintenance of proinflammatory cytokine release through interconnected feedback loops in patients with severe symptomatology, our model predicts that IL-10 induces proinflammatory molecules, including IL-18, FN1, PLAU, and VCAN, in a subpopulation of macrophages, despite its known role as an anti-inflammatory cytokine (*23*). IL-10 signaling has been shown to suppress proinflammatory cytokine expression through the activation of the signal transducer and activator of transcription 3 (STAT3). In addition, it also can activate STAT1 and STAT5 (*24*). Because of the induced expression of proinflammatory cytokines in our model, we investigated which downstream TFs are activated by IL-10 signaling in our model and observed that only STAT1, but not STAT3, is activated in macrophages. This finding is consistent with a previous study demonstrating that IL-10 is reprogrammed toward STAT1 induction in the presence of IFNG, which establishes a proinflammatory gene expression profile (*25*). To further support this finding, we investigated whether known proinflammatory target genes of IL-10 signaling are elevated in this subpopulation compared to patients with mild symptoms where IL-10 signaling activates both STAT1 and STAT3. We observed a statistically significant increase in the expression of IL-6, CXCL8, vascular endothelial growth factor, and IL-17R (*P* < 6.58 × 10^−7^), the major targets of IL-10 (*25*, *26*). Together, this indicates that IL-10 signaling contributes to the establishment of a proinflammatory gene expression profile in a subpopulation of macrophages by activating STAT1, but not STAT3.

### Modulating inflammation in patients with severe COVID-19 symptomatology

After characterizing the molecular differences and commonalities in patients with mild and severe COVID-19 pathologies, we set out to identify potential target genes for modulating the immune response. Therefore, we sought to identify genes that could be targeted for modulating the immune response in patients with severe symptomatology. On the basis of the identified positive feedback loops in patients with mild and severe symptomatology, we simulated the effect of inhibiting ligands and receptors, by manually removing each gene individually from the positive feedback loops. Then, we ranked the perturbed genes by their ability to disrupt feedback loops unique to severe disease cases (see Materials and Methods for details). As a result, our simulation identifies VCAN and TLR2 as the top-ranking target genes whose inhibition disrupts 76 and 75% of the interconnected feedback loops unique to severe cases, respectively, while not interfering with immune response mechanisms common to both cases (Fig. 4C). In particular, the maintenance of IL-6, IL-18, IL-15, and IL-27 and the induction of proinflammatory cytokines by IL-10 are fully disrupted according to our model, while the anti-inflammatory, autocrine feedback of IL-21 in regulatory T cells remains intact (Fig. 4D). However, to date, no approved compound inhibiting VCAN exists. In addition, adapalene has been identified as an inhibitor of TLR2. However, its current application form (topical administration) is not readily suitable for patients with COVID-19.

Last, we aimed at validating the predicted molecules using two independent bronchoalveolar lavage fluid samples from German patients with severe symptomatology (*27*). Using our methodology, we reconstructed the positive feedback loops and compared it against the previously detected positive feedback loops of patients with mild symptomatology. As a result, TLR2 and VCAN were able to disrupt the fifth and sixth most feedback loops, only exceeded by CCR6, its cognate ligand CCL20, IL-2RG, and syndecan 1. In contrast to the cell-cell interactome of the German patients where the interaction between CCL20 and CCR6 is present, it could not be detected in the cell-cell interactomes of Chinese patients, since the receptor was not significantly associated with the expression of downstream TFs, which warrants the comparison to samples from patients with mild symptoms having the same genetic background.

## DISCUSSION

In this study, we proposed a computational model for predicting immunomodulatory compounds and target proteins to treat severe symptomatology in patients with COVID-19. The model integrates both intra- and extracellular signaling interactions with gene regulatory networks. Unlike current strategies for identifying potential immunomodulatory proteins and compounds, our method detects and exploits the amplifying feedback loops governing the dysregulated inflammatory response, thereby providing a holistic view of the extracellular cell-cell communication network underlying the disease pathology. In addition, not only the extracellular signaling is modeled by evidence-based cognation of ligand and receptors, but it is also further scrutinized in light of the compatibility with downstream intracellular signaling cascades. In contrast to current strategies for predicting immunomodulatory proteins and compounds, to our knowledge, this is the first method incorporating molecular information about inflammatory processes.

The proposed methodology relies on positive feedback loops, which play a key role in the amplification of the immune response to pathogenic factors (*8*, *9*). Although positive feedback loops can be observed in physiological immune reactions, a runaway inflammation is prevented through the establishment of negative feedback loops (*28*). In contrast, pathological inflammation is characterized by the presence of positive feedback loops whose amplification is insufficiently restricted by negative feedback loops. Therefore, it is expected to find positive feedback loops under both conditions. In this regard, the proposed strategy of targeting positive feedback loops unique to pathological immune responses constitutes a rational approach for modulating inflammation, since it disrupts loops that are newly established and amplified because of the absence of sufficient negative feedback.

The validity of our method was further corroborated by predicting immunomodulatory proteins and compounds targeting them in the context of 12 diseases characterized by a pathological immune response. In particular, we were able to validate 93% of the top-ranking proteins with previous studies highlighting the efficacy of targeting these predicted genes. In the context of COVID-19, using our method, we were able to identify VCAN and TLR2 as potential targets for immunomodulatory attempts, which was further confirmed by analyzing two independent patients with severe symptomatology. VCAN is an extracellular matrix glycoprotein, which creates a strongly adhesive environment for monocytes and T cells (*29*, *30*). In response to an acute inflammation, VCAN accumulates in the extracellular matrix of the inflamed tissue leading to accumulation of leukocytes (*31*). Previous studies showed that interference with VCAN significantly dampens virus-induced inflammation and CCL2-induced monocyte migration (*32*). We hypothesize that the overexpression of VCAN in severe disease cases results in the excessive accumulation of proinflammatory monocytes in the lung, which is further supported by an increased number of monocytes. Thus, VCAN constitutes a plausible, novel target gene for modulating the hyperinflammatory response in patients with COVID-19 with severe symptomatology.

Despite the demonstrated ability of our method to predict immunomodulatory proteins and compounds, it has limitations. Namely, it requires single-cell RNA-seq data of tissues displaying pathological and physiological immune responses, which is currently not widely available. However, the steadily increasing availability of single-cell technologies and publicly available single-cell RNA–seq datasets is expected to alleviate this issue in the future. In addition, our method focuses on the effect of ligand-receptor–mediated cell-cell communication. Nevertheless, other signaling mechanisms exist that are important for establishing a proper inflammatory response, such as through the exchange of exosomes (*33*), which could extend the current scope of this method. Last, accurate predictions of our method require the correct clustering and annotation of the input data. Although the inherent heterogeneity in single-cell data is an advantage in the detection of strong cell-cell interactions, artificial heterogeneity as a result of inaccurate cell type identification is a notable impediment to the detection of feedback loops. In particular, the association of ligands to causally dependent receptors inducing their expression requires the presence of a sustained regulatory path, which is crucially dependent on the cell type each cell is associated with.

In summary, the presented method provides the first strategy to systematically identify immunomodulatory proteins and the compounds targeting them. Thus, we believe that it will be of great utility in the characterization of pathological immune responses and in the design of novel therapeutic interventions for a wide range of diseases associated with exuberant or persistent inflammation, including COVID-19.

## MATERIALS AND METHODS

### Single-cell RNA-seq data processing

Single-cell RNA-seq datasets were obtained from publicly available databases (table S2). Whenever possible, datasets were obtained in processed form. In case this was not possible, each dataset was processed according to the guidelines in the original studies reporting them. Cell type identification for COVID-19 samples of German patients was performed by transferring the labels of COVID-19 samples of Chinese patients using Seurat’s “TransferData” function (*34*, *35*).

### Assembly of cell-cell communication scaffolds, intracellular signaling, and TF-gene regulatory interactions

A cell-cell communication scaffold was generated by manual curation of PPIs contained in iRefIndex (version 16, 09.10.2019) (*36*) and in a previously published dataset (*37*). PPIs from iRefIndex were selected that showed taxon ID “9606,” interaction type “MI:0407 (Direct Interaction)” and contained an HGNC (HUGO Gene Nomenclature Committee) symbol information for both interactors. Furthermore, the interaction had to involve one ligand and receptor, respectively, based on the definition provided in the Cell-Cell Interaction Database (*38*, *39*). An interaction was included in the dataset if the binding occurred extracellularly.

The intracellular signaling network is composed of pathway interactions included in OmniPath (*40*), Reactome (*41*), or MetaCore from Thomson Reuters. In particular, all pathways from MetaCore were obtained including all signal transduction interactions while discarding transcriptional gene regulatory interactions.

Gene regulatory interactions were obtained from MetaCore from Thomson Reuters, a manually curated resource of gene-gene interactions, on 01.04.2019 for human genes. Only transcriptional regulatory interactions with known effects, i.e., activation or inhibition, were selected by filtering for “direct interactions” with reported effects “activation” or “inhibition.”

### Inference of the cell-cell interactome

The main algorithm consists of four steps. First, preserved TFs of each cell type are detected. For that, the method discretizes the expression matrix, such that (non)zero counts become “1” (“0”), respectively, and selects TFs that are expressed in (i) at least 5% of cells of a given cell type and (ii) the 95 percentile of cells.

Second, preserved TFs are connected to the receptors inducing their expression using a Markov chain model of intracellular signaling, called SigHotSpotter (*42*). SigHotSpotter relies on single-cell RNA-seq data of a single cell type and an intracellular signaling network to simulate the traversal of an extracellular signal through the network as a discrete time Markov chain. Genes with the highest steady-state probability are selected and defined as being active or inactive depending on their compatibility with downstream, preserved TFs. Compatibility is determined by assessing the sign of all shortest paths between an intermediate molecule and its downstream TF targets. A path is considered activating if it consists an even number of inhibitions and inhibiting otherwise, such that both the intermediate and downstream TFs have to be expressed in case of an activating path and not expressed in case of an inhibiting path. An intermediate molecule is considered compatible if a significant number of its target is compatible and assessed with a hypergeometric test (*P* cutoff = 0.05). Following the same rationale, receptors are identified that target intermediate molecules, which establishes a connection between receptors and preserved TFs.

Third, ligands expressed in at least 5% of cells of each cell type are selected. Last, ligand-receptor interactions are established between two cell populations if (i) the receptor was selected in the first step for the first population, (ii) the ligand was selected in the second step for the second population, and (iii) the receptor-ligand interaction is contained in cell-cell communication scaffold. Every interaction is augmented with an interaction strength defined as the product of the average receptor expression and average ligand expression in all cells of a population expressing the receptor or ligand, respectively. Significance of each interaction is determined on the basis of the scores of all cell-cell interactions in the cell-cell communication scaffold between the two interacting populations. Scores in the top quantile are considered significant.

### Detection of positive feedback loops

Causal feedback loops were inferred following three steps. Initially, we linked each receptor in the cell-cell communication network to its coexpressed ligands in each cell population having a conserved regulatory path from the receptor. For that, we first transformed the expression data into binary values as described for the selection of preserved TFs. Then, significantly coexpressed ligands and receptors are detected by comparing the fraction of cells agreeing in binary receptor and ligand expression to a random distribution composed of coexpression values of randomly paired ligands and receptors in the same population. A *P* value below 0.05 is deemed significant. Eventually, conserved shortest paths between the receptor and its coexpressed ligands are calculated on the TF-gene interactomes (see above) using igraph (*43*). In particular, starting from TFs being targeted by the receptors in the cell-cell interactomes, the fraction of cells in a subpopulation sharing a shortest path to a coexpressed ligand is computed when the receptor is active and inactive, respectively. A user-defined threshold for the minimum (maximum) fraction of cells having a conserved path when the receptor is active (inactive) links receptors to ligands intracellularly. Here, minimum and maximum thresholds of 0.4 and 0.25 were used, respectively.

To identify causal feedback loops, we combined cell-cell interactions and intracellular links between receptors and the coexpressed ligands in a global network representation using igraph (*43*).

### Identification of target proteins and compounds

Using the network of identified positive feedback loops under two conditions, the method computes potential target proteins defined as being unique to the feedback loops of the pathologic condition. Each potential protein is scored as follows: First, the number of ligands participating in pathological feedback loops is computed on the basis of a manually assembled list. Subsequently, the strongly connected components of the pathological feedback loop graphs are computed after removing the potential target gene. Last, the fraction of removed ligands within strongly connected components is computed, which serves as the score for the protein. Compounds inhibiting the top-scoring proteins are obtained from a list of drugs and their inhibited target proteins originally retrieved from DrugBank (*16*).

## SUPPLEMENTARY MATERIALS

Supplementary material for this article is available at http://advances.sciencemag.org/cgi/content/full/7/6/eabe5735/DC1

## Appendix

View/request a protocol for this paper from *Bio-protocol*.
